# Low-volume transanal irrigation (TAI) in the treatment of functional faecal incontinence in children: a cohort study

**DOI:** 10.1007/s00384-025-04813-0

**Published:** 2025-01-30

**Authors:** Nicklas B. Hougaard, Rene F. Andersen, Konstantinos Kamperis, Cecilie S. Jørgensen

**Affiliations:** 1 Department of Paediatrics and Adolescent Medicine, Aarhus University Hospital, Palle Juul-Jensens, Boulevard 99, 8200 Aarhus N, Denmark; 2 Department of Clinical Medicine, Aarhus University, Palle Juul-Jensens Boulevard 99, DK-8200 Aarhus N, Denmark

**Keywords:** Functional constipation, Functional faecal incontinence, Transanal irrigation

## Abstract

**Purpose:**

Functional faecal incontinence (FFI) is a stigmatising condition for a child and parents and can be a challenge to treat even in tertiary centres. Transanal irrigation (TAI) is an emerging treatment with great success in refractory cases. We performed TAI with a substantially decreased amount of water used (low-volume TAI), yet no previous evidence exists on this treatment in children. We conducted this study to evaluate the efficacy of low-volume TAI in reducing faecal incontinence (FI) episodes and to provide associated factors for response.

**Methods:**

Children with FFI trained in low-volume TAI in our outpatient incontinence clinic were identified. Baseline characteristics along with rectal ultrasound examination, information on weekly FI episodes and concomitant use of laxatives were noted. The continence status of patients was registered at the first outpatient clinic appointment after the commencement of TAI and after 6 months of treatment. During this period, information about side effects and changes in medication were captured.

**Results:**

We identified 47 children (mean age 8.06 ± 2.08 years, 27 males) treated with low-volume TAI. Thirty-five (74%) were diagnosed with functional constipation and FI, while 12 (26%) suffered from non-retentive faecal incontinence. Twenty (42%) children gained full faecal continence after 6.75 ± 0.3 months. We found no differences between responders and non-responders in baseline characteristics.

**Conclusions:**

Low-volume TAI appears safe and effective in the treatment of FFI refractory to first-line treatment in children. Low-volume TAI could be a valuable tool for the management of these children as the treatment is less invasive, low in cost and well accepted.

## Introduction

Functional faecal incontinence (FFI) in children is a stressful condition for both the affected child and the parents and is associated with reduced quality of life [1, 2]. Functional constipation (FC) in children defined by the Rome-IV criteria [3] is reported with a pooled prevalence of 9.5% in a recent meta-analysis [4]. Up to 75–90% of these children also report episodes of faecal incontinence (FI) [5]. When faecal retention and relevant medical conditions are absent, incontinence episodes are termed functional non-retentive faecal incontinence (FNRFI) [3]. FNRFI is estimated to account for 10–30% of cases with FFI [6].

Transanal irrigation (TAI) is widely used in adult and paediatric neurogenic bowel disease to promote faecal continence [7–9]. TAI in children refers to a volume of irrigant of 10 to 20 ml/kg [10] often resulting in volumes ranging from 300 to a maximum of 1000 ml [11]. Accordingly, in this setting the approach can be termed high-volume TAI. During the last decade, high-volume TAI has gained recognition in the treatment of FC and FNRFI and currently plays an important role before subjecting treatment refractory FI children to surgical procedures, where response is still not guaranteed [6, 11]. Treatment with high-volume TAI has significant efficacy on reducing FI episodes thus leading to an increase in quality of life, and the child and parent treatment satisfaction remains high [2, 11–14]. At our centre, TAI has since 2010 been successfully used with complete remission seen in 73% of children with FFI [14].

During recent years, some children with treatment refractory FI at our centre have been treated with a low-volume TAI prior to additional medical treatment attempts and/or high-volume TAI. Research on low-volume TAI is limited and data is only available in adults [15]. Low-volume TAI uses a volume ranging from 50 to 200 ml of irrigation and might be more acceptable to children than high-volume TAI. Furthermore, the treatment is less invasive, cheaper and requires less time for patients, parents, and healthcare providers.

We conducted this study with a hypothesis that low-volume TAI is effective in children with FI in terms of reducing FI episode frequency. The aim of the study was to determine the effect of low-volume TAI in obtaining faecal continence and to provide associated factors for response.

## Methods

### Patients and materials

We performed a cohort study at the Centre for Childhood Incontinence, Department of Paediatrics and Adolescent Medicine, Aarhus University Hospital, Denmark, a tertiary referral centre for childhood incontinence. Patients treated with low-volume TAI were included from 2016 to 2021. Children were excluded if they had neurogenic bowel disease, anorectal malformations, Hirschsprung’s disease or if they had used high-volume TAI before or during treatment with low-volume TAI. All children were characterized as having constipation-associated (retentive) FI or non-retentive FI based on the Rome-IV criteria for FC and FNRFI [3], and transabdominal rectal ultrasound and colon transit time if performed. Hence the exclusion of non-functional FI children, retentive and non-retentive FI will in the following be synonymous with functional disorders. Patients were allowed to continue the use of laxatives during treatment with TAI (macrogol 3350, magnesium oxide, sodium picosulfate and/or rectal docusate or bisacodyl) as well as treatment for urinary incontinence. Changes in medication were noted.

### Treatment approach

At our centre, first-line treatment of FI in children consists of a conservative and pharmacological approach. The conservative part of the treatment comprises education of the child and parents including structured toilet training. If the child presents with retentive FI, laxative treatment with macrogol 3350 is initiated and, if unsuccessful, followed by treatment attempts with magnesium oxide, sodium picosulfate and/or rectal docusate or bisacodyl enemas. If the child presents with non-retentive FI, rectal enemas with docusate or bisacodyl can be tried. In the case of persistent FI episodes, further investigations as colonic transit time can be indicated. This approach is in accordance with published guidelines in managing retentive and non-retentive FI [16, 17]. In our clinical setting, children normally receive low-volume TAI if they are refractory to standard laxative treatment or have side effects to treatment with rectal docusate or bisacodyl enemas.

### Low-volume transanal irrigation

Low-volume TAI was performed using a rubber enema spray with a soft cannula (SoftWash, Pic Solution), able to hold a volume of up to 115 ml. Depending on the age and weight of the child, a daily volume of 50 to 200 ml was used. Administration was performed twice if volume exceeded the volume of the enema. Patients received instruction to sit on a toilet, administer the low-volume TAI containing tap water at about 37 °C and remain seated on the toilet for 10–15 min to allow evacuation of stools and water. Some patients were able to self-administer the TAI. Follow-up was individually scheduled and decided by the physician resulting in varying time between visits. If the response was obtained the patient was instructed in carefully skipping days of irrigation. If no response was obtained the volume could be increased to a maximum of 200 ml.

### Outcome measures

The primary outcome measure was the number of FI episodes per week. This variable was obtained from the record of outpatient clinic visits at the commencement of low-volume TAI and continuously through the treatment period. Full response was defined as complete remission of FI episodes and partial response was defined as more than fifty percent reduction in FI episodes analogically with terminology used in lower urinary tract function defined by The International Children’s Continence Society [18]. In the following analysis, responders will be synonymous with children obtaining a full response, whereas non-responders cover children with partial or no response. Relapse episodes were noted. Outcome measures were recorded at the time of discontinuation of treatment or at the last follow-up.

### Statistical analyses

Categorical variables are presented as frequency and percentage. Continuous data were tested for normal distribution by histogram and Q-Q-plot and presented as the median and interquartile range (IQR) if non-normally distributed or mean and standard deviation (SD) if normally distributed. Test of the hypothesis was made using Fischer’s exact test for categorical values and Student’s *t*-test for normally distributed numeric data. Significance was considered when *p* < 0.05.

Statistical analyses were conducted using Stata, version 17.0 (StataCorp, LCC).

### Ethics approval

The study was carried out as a study of quality improvement. According to the Danish Health Care Act, this type of study is without the need for obtaining formal consent if the patient has had contact with the investigating department within the last 5 years. Furthermore, no ethical approval is required. Only non-identifiable information was retrieved from the electronic patient journal.

## Results

### Patient characteristics

We included 47 children with a mean age of 8.06 ± 2.08 years. Table 1 summarizes the overall characteristics of patients stratified by incontinence type. Overall, 27 (57%) children were boys, and eight (17%) had a psychiatric comorbidity of attention-deficit/hyperactivity disorder and/or autism spectrum disorder. Twenty-two (47%) children suffered from urinary incontinence as well. Two-thirds of the patients used oral or rectal-administered laxatives at the commencement of low-volume TAI. Twenty-eight (60%) children used macrogol 3350, seven (15%) children used sodium picosulfate, and seven (15%) children used rectal docusate or bisacodyl enemas. Thirty-five (74%) children met the Rome-IV criteria for FC and had FI while twelve (26%) thereby per definition were classified as FNRFI [3]. The rectal diameter measured by ultrasound differed nearly significantly between the groups (3.1 ± 0.89 cm vs 2.41 ± 0.63 cm, *p* = 0.07). The use of laxatives was substantially higher in retentive FI compared to non-retentive FI (86% vs. 17%, *p* < 0.001).

**Table 1:** Baseline patient characteristics: overall and children with FC-related faecal incontinence (retentive FI) vs. children with functional non-retentive faecal incontinence (non-retentive FI). Data presented as n (proportion) or mean (SD)

	Overall	Retentive FI	Non-retentive FI	*p*-value
*n*	47	35	12	-
Sex (male)	27 (57%)	19 (55%)	8 (67%)	.55
Age (years)	8.1 (2.08)	7.8 (2.12)	8.9 (1.74)	.10
Psychiatric comorbidity*	8 (17%)	6 (17%)	2 (17%)	> .99
FI episodes before low-volume TAI, weekly				
1–3	17 (36%)	11 (31%)	6 (50%)	.31
4– > 8	30 (64%)	24 (69%)	6 (50%)	
Rectal diameter (cm)	2.94 (0.88) *Missing* = *36%*	3.10 (0.89) *Missing* = *34%*	2.41 (0.63) *Missing* = *41%*	.07
Laxative use**	32 (68%)	30 (86%)	2 (17%)	** < .001**
Urinary incontinence				
Daytime	19 (40%)	14 (40%)	5 (42%)	> .99
Enuresis	14 (30%)	9 (26%)	5 (42%)	.47

*Psychiatric comorbidity: attention-deficit/hyperactivity disorder and autism spectrum disorder

**Laxatives: macrogol 3350, magnesium oxide, sodium picosulfate and/or rectal docusate or bisacodyl

### Response to treatment

Twenty (42%) children gained full faecal continence after a median treatment duration of 6.75 ± 0.3 months (Table 2). Of these, thirteen (28%) children experienced a full response already after the first outpatient visit post-commencement of low-volume TAI. The median time to the first outpatient visit was 1.87 ± 0.65 months. At the time of the first outpatient visit, seven (15%) children were lost to follow-up or had terminated the use of low-volume TAI. At the endpoint of the study, this group was increased to a total of fourteen (30%) children.

**Table 2:** Response registered at the first outpatient visit after commencement and after 6 months of treatment. Data presented as n (proportion) or mean (SD)

	Full response	Partial or none response	Terminated use	Time to outpatient visit, months
First outpatient visit after commencement	13 (28%)	27 (57%)	7 (15%)	1.87 (0.65)
Outpatient visit after 6 months	20 (42%)	13 (28%)	14 (30%)	6.75 (0.30)

Table 3 presents baseline characteristics stratified by response to treatment. There were no significant predictors for response when examining age, pre-treatment FI episodes, psychiatric co-morbidity, pre-treatment rectal diameter, laxative use, or coexistence of urinary incontinence.

**Table 3:** Characteristics of responders (full response) vs. non-responders (partial or no response and terminated use). Response was registered after 6 months of treatment. Described as n (proportion) unless otherwise stated

	Responders	Non-responders	*p*-values
*n*	20 (42%)	27 (58%)	-
Gender (male)	10 (50%)	17 (63%)	.55
Age (years), mean (SD)	8.4 (2.6)	7.8 (1.59)	.31
Type of FI			
Retentive FI	16 (80%)	19 (70%)	.52
Non-retentive FI	4 (20%)	8 (30%)	
Psychiatric comorbidity*	5 (25%)	3 (11%)	.26
Episodes before TAI, weekly			
1–3	7 (35%)	10 (37%)	> .99
4– > 8	13 (65%)	17 (63%)	
Rectal diameter, mean (SD)	3.06 (0.86) *Missing* = *35%*	2.85 (.90) *Missing* = *37%*	.53
Laxative use, yes**	14 (70%)	18 (67%)	> .99
Urinary incontinence			
Daytime	9 (45%)	10 (37%)	.76
Enuresis	8 (40%)	6 (22%)	.21
Later use of high-volume TAI	0 (0%)	6 (22%)	.06
Changes of medication	4 (20%)	8 (32%)	.50

*Psychiatric comorbidity: attention-deficit/hyperactivity disorder and autism spectrum disorder

**Laxatives: macrogol 3350, magnesium oxide, sodium picosulfate and/or rectal docusate or bisacodyl

Five (12%) children reported dissatisfaction with the irrigation and discontinued treatment with low-volume TAI. However, only two (4%) children experienced pain and/or soreness when irrigating. No adverse events (e.g., perforation) were registered in the entire period of follow-up.

Data revealed, that four (20%) of the responders and eight (32%) non-responders had changes in their medication within the first 6 months. For non-responders, this included the addition of further laxatives to the treatment, whereas two of the responders paused the use of laxatives and two had changes in the laxative treatment.

## Discussion

The present study shows a relatively high response rate (42%) to low-volume TAI after 6 months of treatment in children with retentive and non-retentive FI refractory to first-line treatments. Only a minor percentage (4%) of children reported pain or discomfort when irrigating. These findings suggest that low-volume TAI could play an important role in the treatment of FI after unsuccessful conservative and laxative treatment and as a precursor to the more demanding high-volume TAI. To our knowledge, this is the first study conducted on low-volume TAI in children with FI. All children included in this study were refractory to standard treatment including laxatives, hence this group of patients is challenging to treat. A study found low age and underlying diagnoses as behavioural disorders to be predictors of poor response to high-volume TAI [2], which was not the case in this study of low-volume TAI.

Scintigraphic evaluations of high-volume TAI show a washout of stools in the entire rectal ampulla and colon descendens, providing continence between irrigations and, by time, regaining of the propulsive ability and control over defecation [19]. In children, high-volume TAI volumes of up to 1 L have been used [11], whereas this study examined a volume of 50—200 ml. Volume was decided depending on the age and size of the child. This difference in volume influences the mechanics of colonic emptying and theoretically provides a less effective wash-out. In a study of low-volume TAI in adults, using a volume of only 70 ml, a high proportion converted from low-volume to high-volume TAI due to unsatisfactory effect [15]. However, our results indicate that a relatively small volume of irrigant might be enough to obtain continence in many children.

We have no follow-up data on a potential relapse of incontinence episodes, however, as in children treated with high-volume TAI, relapse can be expected. In some children, relapse could be explained by an aggressive down titration of low-volume TAI thus gradual reduction is suggested to avoid relapse of FI episodes [11]. Our patients were instructed to gradually reduce in days of irrigation 1–2 months after the response was obtained. Additionally, they were instructed to resume irrigation if a relapse happens. Another explanation could be a more retrograde faecal impaction in FC children that low-volume TAI is unable to dissolve resulting in worsening in the symptoms when down titrating.

Apart from the lower volume used in low-volume TAI, further differences from high-volume TAI are to be noted. Most children in our ambulatory setting report using between 10 and 15 min when using low-volume TAI compared to high-volume TAI, where the normal time easily extends to 20–40 min per irrigation [13]. Children and their parents might be more willing to commence low-volume TAI as it is less intimidating and time-consuming. Furthermore, low-volume TAI could be easier performed by the child without help from the parents as the procedure is simpler and less painful due to the lower volume used. In this study, 4% of children discontinued the use of low-volume TAI due to pain at insertion or irrigation. When using high-volume TAI, up to 42% of children report pain during the procedure [20]. No adverse events occurred during the study period and low-volume TAI seems to be a safe treatment in line with the safety profile of high-volume TAI [21].

The device for low-volume TAI used in this study is a broadly available soft rubber cannula that comes in a variety of volumes. It comes at a very low price (< 10 €) compared to traditional high-volume TAI systems. However, challenges regarding cleanse after use and user-friendliness remain.

Some limitations of this study should be mentioned. Firstly, the nature of the study as a cohort study introduces potential misclassification bias and confounding together with a risk of irretrievable information. Secondly, a challenge in this population was the missing training sessions together with the lack of a protocol for short-time follow-up. Training and follow-up are critical in children receiving high-volume TAI to enhance the rate of success and compliance [11, 22]. If all children had undergone a standardised training protocol and a follow-up regime, there might have been a higher proportion of children commencing low-volume TAI. The question remains if these adjustments would improve the response rate or if the cases described in this paper were the most motivated and thereby most compliant. Furthermore, the small number of patients in this study is a limitation as additional patients could have strengthened the associations and might as well have provided predictive factors.

The information on the use of laxatives was collected at the commencement of low-volume TAI and changes in medication were captured in the first 6 months. Changes in the medication might have influenced the outcome, however, only 20% of the responders had changes in medication during TAI. Long-term medical treatment are essential in handling constipation and FI [10, 23] and one can speculate if the combination of low-volume TAI and laxatives for many children is necessary in order to gain continence.

There is a need for prospective, controlled studies on low-volume TAI in order to rank the treatment in the algorithms for treating FI and FC and to compare low-volume TAI with high-volume TAI, oral and rectal laxatives. Furthermore, it would be of interest to identify associated factors and to examine the use of low-volume TAI in neurogenic FI children, where high-volume TAI is frequently used.

In conclusion, low-volume TAI appears a safe and effective treatment in FFI children refractory to first-line treatment. We identified no significant predictors for response. Low-volume TAI may deserve a place in the treatment of FI children.

## Data Availability

No datasets were generated or analysed during the current study.
